# Editorial Note: Zileuton Improves Memory Deficits, Amyloid and Tau Pathology in a Mouse Model of Alzheimer’s Disease with Plaques and Tangles

**DOI:** 10.1371/journal.pone.0349725

**Published:** 2026-05-20

**Authors:** The PLOS One Editors

The *PLOS One* Editors issue this Editorial Note to inform readers of similarities between images originally published in this article [1] and those in three later‑published articles [2–4] by some of the same authors, all of which have since been retracted.

Specifically, the following similarities were identified between panels with differently labelled samples and/or conditions:

- The AT8 3xTg-zileuton panel in Fig 3D of [1] appears similar to the AT8 htau-zileuton panel in Fig 3F of [2].
- The AT180 3xTg panel in Fig 3D of [1] appears similar to the AT180 htau panel in Fig 3F of [2].
- The MAP2 3xTg panel in Fig 4E of [1] appears similar to the MAP2 WT and MAP2 WT‑zileuton panels in Fig 5C of [3].
- The PSD-95 3xTg‑zileuton panel in Fig 4E of [1] appears similar to the PSD-95 WT-5LO panel in Fig 4C of [4].

The corresponding author states that the correct experimental information is reported in this article [1], which precedes other articles containing duplicate material.
